# Transuterine infection by *Baylisascaris transfuga*: Neurological migration and fatal debilitation in sibling moose calves (*Alces alces gigas*) from Alaska

**DOI:** 10.1016/j.ijppaw.2018.07.005

**Published:** 2018-07-19

**Authors:** Eric P. Hoberg, Kathleen Burek-Huntington, Kimberlee Beckmen, Lauren E. Camp, Steven A. Nadler

**Affiliations:** a School of Veterinary Medicine, Department of Pathobiological Sciences, University of Wisconsin, Madison, WI, 53706, United States; b Alaska Veterinary Pathology Services, 23834 The Clearing Drive, Eagle River, AK, 99577, United States; c Alaska Department of Fish and Game, Division of Wildlife Conservation, 1300 College Road, Fairbanks, AK, 99701, United States; d Department of Entomology & Nematology, University of California, Davis, CA, 95616, United States

**Keywords:** *Baylisascaris transfuga*, Neonatal infection, Transuterine migration, Neural larval migrans, Phylogenetic identification, *Alces alces gigas*

## Abstract

Larval *Baylisascaris* nematodes (L3), resulting from transuterine infection and neural migration, were discovered in the cerebrum of sibling moose calves (*Alces alces gigas*) near 1–3 days in age from Alaska. We provide the first definitive identification, linking morphology, biogeography, and molecular phylogenetics, of *Baylisascaris transfuga* in naturally infected ungulates. Life history and involvement of paratenic hosts across a broader assemblage of mammals, from rodents to ungulates, in the transmission of *B. transfuga* remains undefined. Neural infections, debilitating young moose, may seasonally predispose calves to predation by brown bears, facilitating transmission to definitive hosts. Discovery of fatal neurological infections by L3 of *B. transfuga* in mammalian hosts serves to demonstrate the potential for zoonotic infection, as widely established for *B. procyonis,* in other regions and where raccoon definitive hosts are abundant. In zones of sympatry for multi-species assemblages of *Baylisascaris* across the Holarctic region presumptive identification of *B. procyonis* in cases of neurological larval migrans must be considered with caution. Diagnostics in neural and somatic larval migrans involving species of *Baylisascaris* in mammalian and other vertebrate hosts should include molecular-based and authoritative identification established in a phylogenetic context.

## Highlights

• Larval *Baylisascaris* nematodes, demonstrating transuterine infection, were discovered in sibling moose calves from Alaska.
• *Baylisascaris transfuga* was identified through morphology, biogeography and molecular phylogenetics.
• Neural infections in ungulate paratenic hosts may predispose calves to predation by brown bears facilitating transmission.
• Diagnostics for L3 *Baylisascaris* in zones of sympatry for multiple species should include molecular-based identification.

## Graphical abstract

**Figure:**
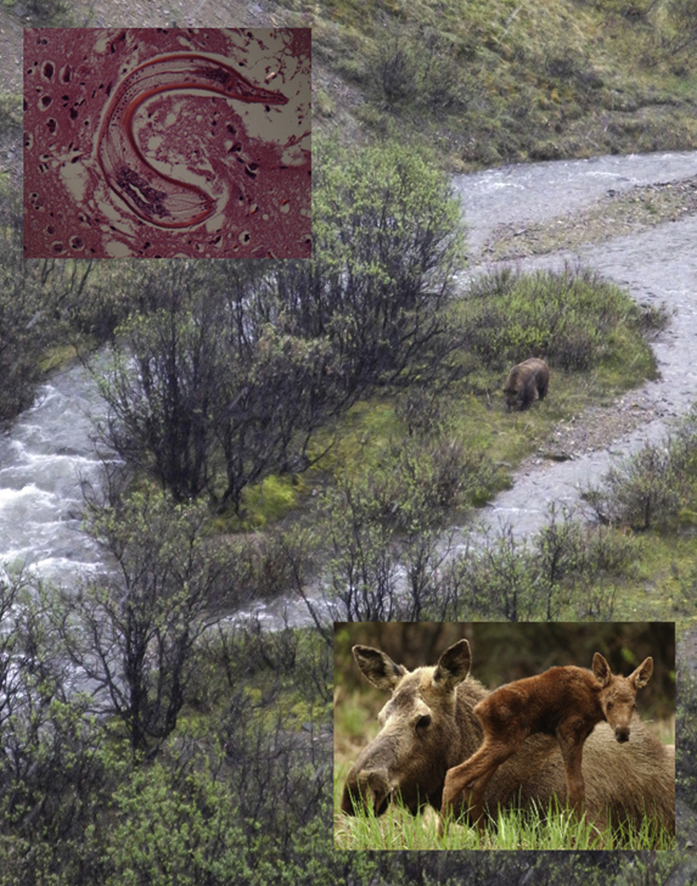

## 1 Introduction

The significance of infections attributed to ascaridoid nematodes among potential intermediate or paratenic hosts is in a propensity for somatic, visceral and neural migration by infective third stage larvae (L3) leading to significant disease and mortality (e.g., Sprent, 1952; Tiner, 1953; Kazacos, 1997, 2001; 2016; Papini et al., 1996; Gavin et al., 2005). Among this assemblage of nematodes neural migration is perhaps best exemplified by L3 among species of *Baylisascaris* Sprent, 1968 and especially *Baylisascaris procyonis* (Stefanski and Zarnowski, 1951). A large nematode of raccoon [*Procyon lotor* (Linnaeus)] endemic in temperate latitudes of North America, *B. procyonis* has also been introduced and established in central Europe and Japan (Okulewicz and Buńkowska, 2009; Beltran-Beck et al., 2012; Robertson et al., 2013; Kazacos, 2016). Although primarily seen in raccoon definitive hosts as adult parasites, the L3 of *B. procyonis* have a broad range of paratenic hosts involved in transmission. Larvae have been documented among a remarkably diverse assemblage of mammals and birds, including reports of significant and devastating zoonotic infections in people (e.g., Kazacos, 1986, 2001; 2016; Anderson, 2000; Gavin et al., 2005; Bauer, 2013; Sapp et al., 2017). In the temperate/boreal zone of North America and areas where *Baylisascaris* has been introduced in Europe or Asia, presumptive diagnosis in cases of neural larval migrans among paratenic hosts has been *B. procyonis* or less often *B. columnaris* (Leidy, 1856). These diagnoses, in the absence of verified vouchers, have been common even in zones of expected sympatry with other congeneric species (reviewed in Kazacos, 2001). The role of true intermediate or paratenic hosts in development and transmission among species of these ascaridoid nematodes in mammalian hosts remains to be clearly defined (Sapp et al., 2017); we adopt the usage of paratenic host in the context of our observations and the following report.

Across the Holarctic and into the Neotropical region, species of *Baylisascaris* are generally typical parasites of medium to large carnivoran definitive hosts including the giant panda with a single species occurring in large rodents (Sprent, 1968; Kazacos, 2001; Zhang et al., 2011; Bauer, 2013). Globally 11 species are recognized, including 4 that are endemic to the Western Hemisphere, 4 species distributed across the Holarctic, 2 endemic to Asia and a single species from Tasmania (Bauer, 2013; Mata et al., 2016; Sapp et al., 2017). Species in carnivores at higher boreal and subarctic latitudes, and also more widely distributed in North America, are represented by *B. transfuga* (Rudolphi, 1819) in brown bear (*Ursus arctos* Linnaeus) and black bear (*U. americanus* Pallas) and *B. devosi* (Sprent, 1952) in medium to large mustelids including weasels and ermine (species of *Mustela* Linnaeus), marten [*Martes americana* (Turton) and *M. caurina* (Merriam)], fisher [*Pekania pennanti* (Erxleben)] and wolverine (*Gulo gulo* Linnaeus) (e.g., Rausch, 1959; Choquette et al., 1969; Hoberg et al., 1990; Borka-Vitális et al., 2017; Sapp et al., 2017). Additional species, including *B. procyonis* in raccoons, *B. columnaris* in skunks (species of *Mephitis* É. Geoffroy Saint-Hilaire and Cuvier and *Spilogale* Gray) and possibly *B. melis* (Gedoelst, 1920) in badgers [*Taxidea taxus* (Schreber)] are currently restricted to more southern, temperate latitudes. The entire assemblage of 4–5 species, however could be in sympatry at some localities in North America (e.g., Pence et al., 1983; Kazacos, 2001; Sapp et al., 2017).

We document and report the occurrence of fatal larval infections involving neural migration by a species of *Baylisascaris* in sibling moose calves (*Alces alces gigas* Miller) from Alaska. Definitive morphological and molecular identification confirmed infection by *Baylisascaris* and in conjunction with recent phylogenetic analyses among species of the genus provided a species level diagnosis (Camp et al. submitted). Neural infections by L3 of *Baylisascaris* in ungulates have been rarely reported and none have been definitively identified at the species level (Anderson, 1999; Kazacos, 2016; Sapp et al., 2017). Further, the current infections clearly represent the outcome of transuterine migration and intrauterine infection. We explore the pathology involved in these cases and the implications for life history patterns and transmission of *Baylisascaris* among bears and moose in Alaska.

## 2 Materials and methods-

### 2.1 Brief history

Twin moose calves from Kincaid Park, a municipal park in Anchorage, Alaska (ca., 61.1541° N and 150.0167° W), estimated to be 1–3 days in age, were orphaned on 23 May 2011 when their dam was shot for aggressive behavior. The calves were subsequently transported to a permitted holding facility maintained by the Alaska Moose Federation, at Palmer, AK. On arrival both calves were wobbly and with wet umbilical cords, indicative of recent birth. The male calf (Alaska Department of Fish and Game, necropsy ID 2011-067 Pathology ID VII-126) was consistently weak and apparently could not suckle or swallow properly, was euthanized on 30 May 2011 and necropsied 24 h later. The female sibling was more vigorous, but later broke her leg, and was euthanized and necropsied on 19 August 2011(Pathology ID VII-201-ADFG OMC ID Tag 56). At necropsy, a panel of tissues was collected for histological evaluation from each calf with specimens fixed in 10% neutral buffered formalin. Intact tissue from the cerebellum and cerebrum of the female calf was also cryo-archived at minus 20 C.

### 2.2 Histology of the nervous system

A suite of tissues including brains were collected for histology and preserved in 10% neutral buffered formalin, processed at Histology Consulting Services (Pullman, Washington), and slides analyzed by a pathologist (KBH). Briefly, paraffin-embedded sections were cut to 5 μm, stained with hematoxylin and eosin and permanently mounted.

### 2.3 Recovery of intact L3 from brain specimen

A frozen, unfixed, tissue sample of several grams from the cerebellum of the infected female calf was received at the US National Parasite Collection in April 2012 (EPH). Tissue was examined in detail initially by compression and transmitted illumination at magnifications to 60×, and secondarily by physical maceration in saline and sieving through a 200 mesh (75 μm) screen. A single larva was recovered from macerated tissue, preserved in 95% ethanol and held at minus 80C for later molecular characterization. Further microscopy of this larval specimen was not conducted given the potential for damage or loss which would have jeopardized attempts for a definitive species-level identification based on DNA sequence comparisons.

### 2.4 DNA extraction and PCR

A presumptive L3 of *Baylisascaris* recovered from the brain tissue was received in the Department of Entomology and Nematology at the University of California, Davis (SN and LEC). This larval specimen was destructively sampled. DNA from a single *Baylisascaris* L3 was prepared using the Epicentre MasterPure Complete DNA Purification Kit (Epicentre Biotechnologies, Madison, Wisconsin). Regions of two mitochondrial genes (12S ribosomal DNA, cytochrome *c* oxidase subunit 2 [*cox2*]), and two nuclear rDNA genes (large-subunit [28S] and the internal transcribed spacers/5.8S gene [ITS-1, 5.8S, ITS-2, abbreviated ITS]) were amplified by PCR. Primers 505 and 506 were used to amplify the 12S region; primers 210 and 211 were used to amplify the *cox2* region. Primers 391 and 501 were used to amplify the 5’ end of the 28S rDNA; and primers 521 and 94 were used to amplify the ITS region. Primer sequences for all genes used for PCR or sequencing are described in Table 1. Other species of *Baylisascaris* and individuals used in the phylogenetic analyses (sequences obtained from GenBank), DNA extraction methods and other procedures are provided in Camp et al. (submitted).

**Table 1:** PCR and sequencing primers.

Gene/vector	Primer name	Direction	Sequence (5′ to 3′)	Reference	PCR (P) or Sequencing (S)
12S	dp505	Forward	GTTCCAGAATAATCGGCTAGAC	Nadler et al., 2006	P and S
	dp506	Reverse	TCTACTTTACTACAACTTACTCCCC	Nadler et al., 2006	P and S
*cox2*	dp211	Forward	TTTTCTAGTTATATAGATTGRTTTYAT	Nadler and Hudspeth, 2000	P and S
	dp210	Reverse	CACCAACTCTTAAAATTATC	Nadler and Hudspeth, 2000	P and S
28S rDNA	dp391	Forward	AGCGGAGGAAAAGAAACTAA	Nadler and Hudspeth, 1998	P and S
	dp501	Reverse	TCGGAAGGACCAGCTACTA	Thomas et al., 1997	P and S
	dp504	Forward	CAAGTACCGTGAGGGAAAGTTG	Nadler et al., 2003	S
	dp503	Reverse	CCTTGGTCCGTGTTTCAAGACG	Nadler and Hudspeth, 2000	S
ITS	dp521	Forward	GTAGGTGAACCTGCGGAAGGATCATT	Gasser et al., 1996	P
	dp94	Reverse	TTAGTTTCTTTTCCTCCGCT	Gasser et al., 1993	P
	dp92	Forward	ATCGATGAAGAACGCAGC	Gasser et al., 1997	S
	dp522	Reverse	GGAATGAACCCGATGGCGCAAT	Zhu et al., 1998	S
pGEM-T	dp617	Forward	CTCCGAACGTGCATAAGCACC	Camp et al., submitted	S
	dp156	Forward	GGCCAGTGAATTGTAATACGACTC	Nadler and Hudspeth, 1998	P and S
	dp157	Reverse	GACACTATAGAATACTCAAGCTATGC	Nadler and Hudspeth, 1998	P and S

For the *Baylisascaris* larva, 25 μl polymerase chain reactions contained 200 μM deoxynucleoside triphosphates, 0.5 units of KOD XL polymerase (EMD Millipore, Billerica, MA), 0.5 μM of each primer, and 2.5 μl of DNA template. For 12S and LSU amplifications, cycling parameters followed Nadler et al. (2006). For *cox2*, cycling parameters followed Nadler and Hudspeth (2000). For ITS, cycling parameters included a 4 min denaturation at 94 °C, then 35 cycles of 94 °C for 30 s, 56 °C for 30 s, 72 °C for 1 min, and a final extension of 7 min at 72 °C.

PCR products were enzymatically treated for sequencing with exonuclease I and shrimp alkaline phosphatase (USB Affymetrix Pre-sequencing kit, USA) and directly sequenced using an ABI 3730 DNA Sequencer (Applied Biosystems, Thermo Fisher Scientific) with the PCR primers (12S, *cox2*), or both PCR primers and internal primers (ITS, 28S, Table 1).

Sequence contigs were assembled using CodonCode Aligner (version 5.1.5, CodonCode Corporation, Centerville, Massachusetts) and Phred base calling. All sequences were double-stranded for verification. Site polymorphisms were recorded only when both alternative nucleotide peaks were present in all sequencing reactions representing both DNA strands. If the heights of the alternative nucleotide peaks at polymorphic sites were not equal, the height of the minor peak was required to significantly exceed background terminations and comprise ≥25% of the major peak to be scored as a polymorphism. GenBank accession numbers for all analyzed sequences are provided in Table 2.

**Table 2:** List of *Baylisascaris* species and outgroups included in analyses.

Species	Host	Collection Location	GenBank Accession # 12S, *cox2*, 28S, ITS
*B. transfuga*	*Ursus americanus*	Alberta, Canada	MH551545, MH551547, MH551546, MH551548
	*Ursus arctos*	Alberta, Canada	MG937792, MH469669, MG937779, MH030601
*B. columnaris*	*Mephitis mephitis*	Connecticut, USA	MG937785, MH469662, MG937772, MH030594
	*M. mephitis*	Illinois, USA	MG937786, MH469663, MG937773, MH030595
*B. procyonis*	*Procyon lotor*	Connecticut, USA	MG937787, MH469664, MG937774, MH030596
	*P. lotor*	California, USA	MG937788, MH469665, MG937775, MH030597
*B. devosi*	*Pekania pennanti*	Ontario, Canada	MG937789, MH469666, MG937776, MH030598
*Baylisascaris* larva	*Alces alces gigas*	Palmer, Alaska	MH509386, MH509388, MH509387, MH509389
*A. suum*	*Sus scrofa domesticus*	Louisiana, USA	MG937795, MH469672, MG937782, MH030604
*P. equorum*	*Equus ferus caballus*	Louisiana, USA	MG937796, MH469673, MG937783, MH030605
*T. leonina*	*Vulpes vulpes*	South Dakota, USA	MG937797, MH469674, MG937784, MH030606

### 2.5 Phylogenetic analysis

*Multiple Alignment*: For non-coding genes (12S, 28S, ITS) sequences for each species or geographic isolate were aligned using ProAlign v0.5a0 (Löytynoja and Milinkovitch, 2003). Nucleotide sequences of the protein-coding gene *cox2* were translated using CodonCode Aligner and the longest open reading frame was selected for each sequence and verified by BLAST comparison. The inferred amino acid sequences for each gene were aligned using default options in CLUSTAL X (Larkin et al., 2007), and the *cox2* nucleotide sequences were aligned to their respective aligned amino acids using the RevTrans Server (v1.4; Wernersson and Pedersen, 2003).

*Parsimony analyses*: Maximum parsimony (MP) analyses were done for each gene and the combined data using the branch-and-bound option of PAUP* version 4.0a150 (Swofford, 2003). To assess clade support values, bootstrap parsimony searches were done using branch-and-bound with 1000 replicates. PAUP* was also used to obtain distance matrices for each aligned gene.

*Bayesian analyses*: Bayesian inference (BI) was done for each gene and the combined data using MrBayes v3.2.6 (Ronquist et al., 2012) executed on the Cyberinfrastructure for Phylogenetic Research (CIPRES) web portal (Miller et al., 2010; http://www.phylo.org). For non-coding genes, best-fit evolutionary models (Table 3) were chosen prior to Bayesian analyses using MrModelTest v2.3 (Nylander, 2004) and the Akaike Information Criterion (AIC). For *cox2*, partitioning schemes and evolutionary models were selected using PartitionFinder v1.1.1 and the AIC (Lanfear et al., 2012). These models were applied to each gene or partition, including the combined analysis. Two independent Bayesian runs were conducted for each analysis with 4 Markov Chain Monte Carlo chains and 4 million generations. Chains were sampled every 4000 generations, and the first 25% of trees were discarded as burn-in. In the post burn-in samples, stationarity was assessed based on: an average standard deviation of split frequencies below 0.01; an average potential scale reduction factor of 1.000; and mean marginal likelihoods that were similar for both runs. Trees remaining after burn-in were used to create 50% majority-rule consensus trees with posterior probabilities for each clade.

**Table 3:** Best-fit evolutionary models chosen for each gene based on MrModelTest v2.3 (12S, 28S, ITS) or PartitionFinder v1.1.1 (*cox2*).

Gene	Model
12S	GTR + G
28S	HKY + G
ITS	HKY + I
Position 1 *cox2*	HKY + I
Position 2 *cox2*	HKY + I
Position 3 *cox2*	HKY + G

## 3 Results

### 3.1 Morphological identification and histology

A full set of tissues were examined microscopically from each calf. Only the pertinent tissue findings are reported respectively for each calf.

**Fig. 1:**
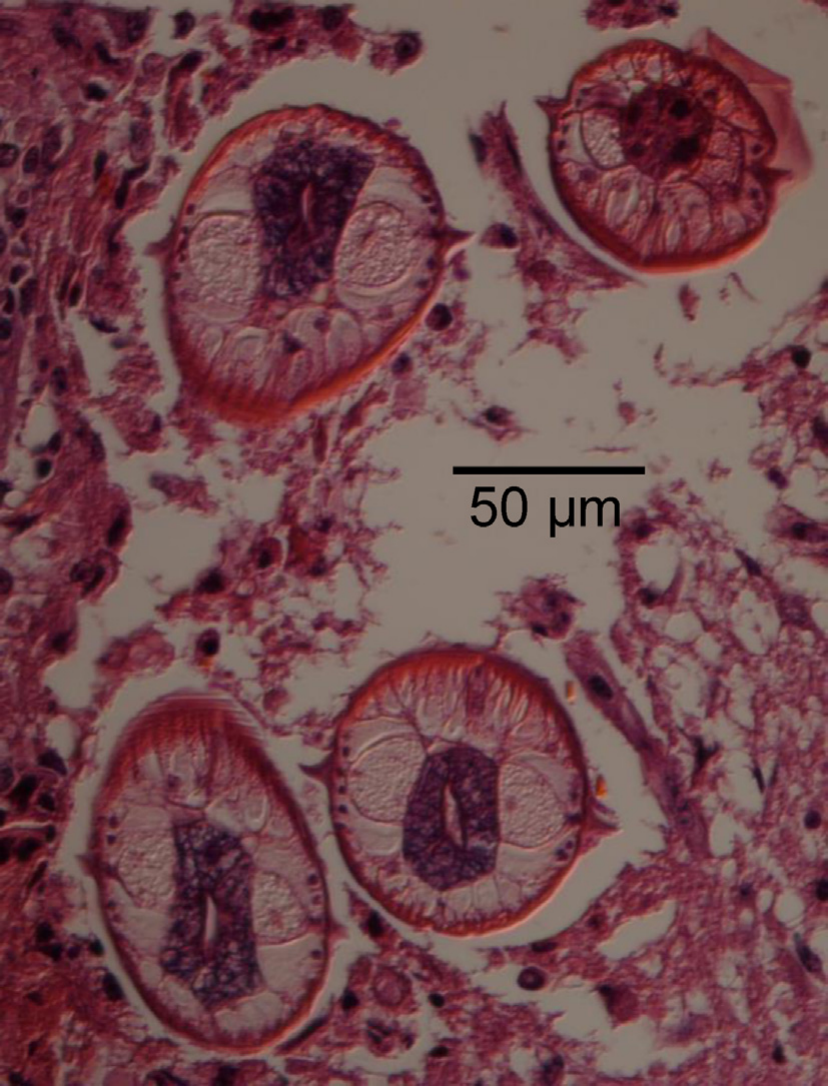
Third stage larvae of *Baylisascaris transfuga* in histological sections of brain of female moose calf (USNPC 108284/Alaska Department of Fish and Game OMC ID Tag 56 Alaska V-11-201); scale = 50 μm. Fig. 1. Brain tissue with L3's in transverse sections. Note prominent lateral alae, coelomyarian polymyarian musculature and morphology consistent with *Baylisascaris*; maximum diameter of L3, 85 μm.

**Fig. 2:**
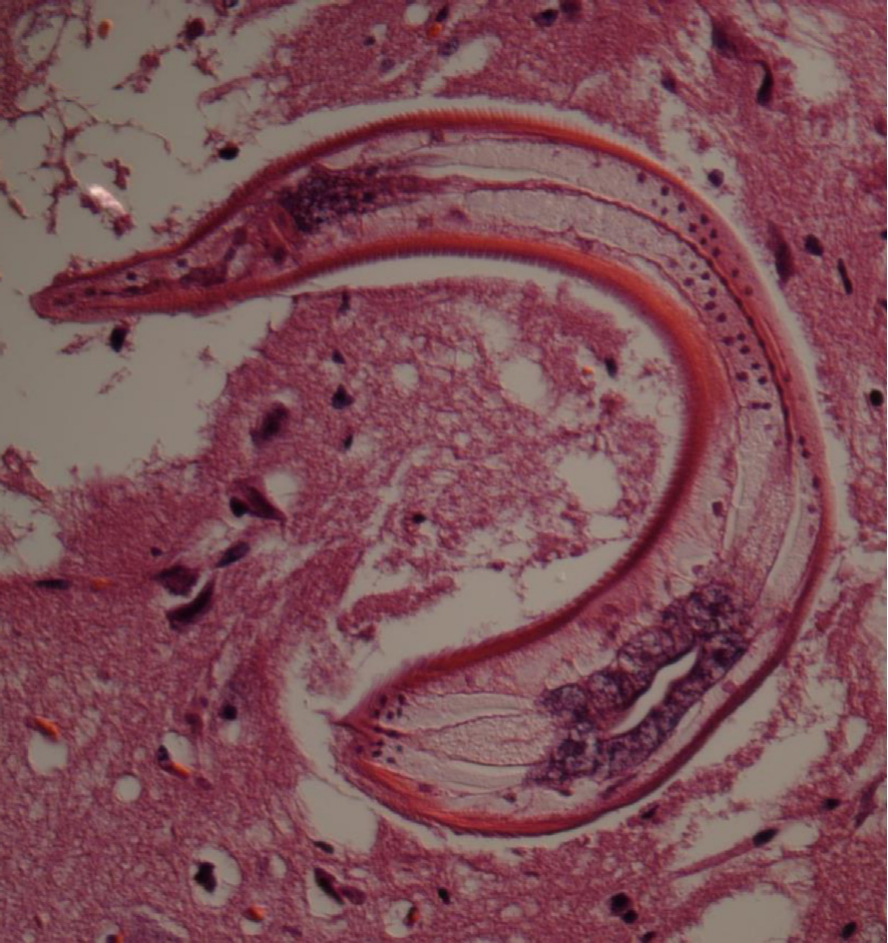
Third stage larvae of *Baylisascaris transfuga* in histological sections of brain of female moose calf (USNPC 108284/Alaska Department of Fish and Game OMC ID Tag 56 Alaska V-11-201); scale = 50 μm. Fig. 2. Third stage larva in longitudinal section, view of cephalic region in brain tissue.

VII-126: Spinal cord, forebrain, pituitary, hippocampus, cerebellum, pons, Circle of Willis and maxillary ganglion were within normal limits. The cerebral cortex contained scattered foci of lymphocytes and gitter cells. In one of these foci, there were also multinucleated giant cells as well as cross sections of parasites. These parasites had a coelom, coelomyarian polymyarian musculature primarily in the dorsal and ventral fields, a bacillary body and prominent lateral alae and a well developed intestinal tract (Fig. 1, Fig. 2). The histopathologic diagnosis was moderate, multifocal random, lymphohistiocytic to granulomatous encephalitis with intralesional nematodes.

V11-201: The eye, spinal cord, pituitary, cerebellum, right anterior hypothalamus, left anterior hypothalamus, and left posterior hippocampus were within normal limits. The right frontal lobe and right posterior hippocampus contained focal pyogranulomatous inflammation around nematode cross sections with features identical to those described above (Fig. 1, Fig. 2). The right posterior hippocampus had a glial nodule with lymphoplasmacytic surrounding inflammation but no foreign body evident. The morphologic diagnoses in both was moderate, multifocal random, lymphohistiocytic to granulomatous encephalitis with intralesional nematodes.

The initial diagnosis in both sibling calves was verminous encephalitis attributed to larval (third stage) ascaridoid nematodes present in the hippocampus and cerebrum. Characteristic third-stage larvae, demonstrated in histology from both calves, were consistent morphologically with a species of *Baylisascaris* (Fig. 1, Fig. 2). In transverse section diameter ranged from 65 to 85 μm. Morphometric data and observations of morphology of the intact L3 from brain tissue were not determined at the time of collection, nor prior to molecular analyses. Voucher histological slides from respective sibling hosts were originally deposited in the US National Parasite Collection (USNPC 105409, single slide from male calf; and USNPC 108284, 2 slides from female calf) which had been curated at the Beltsville Area Research Center of USDA, Agricultural Research Service. Materials are now held in the National Museum of Natural History, Smithsonian Institution, Washington, D.C.

### 3.2 Molecular characterization

**Fig. 3:**
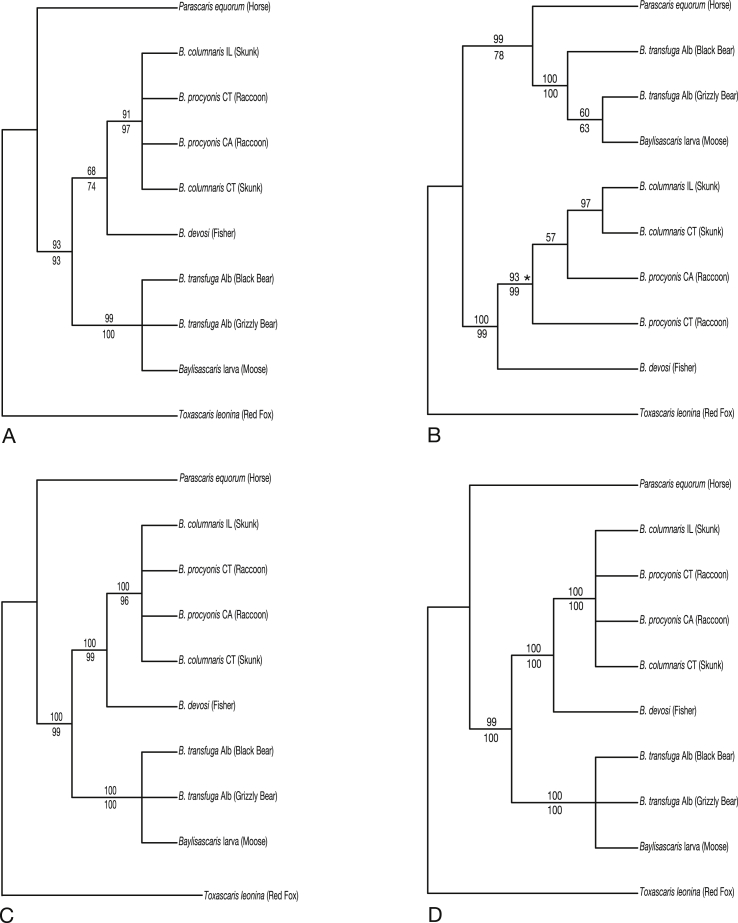
Molecular phylogenetic analyses establishing identity of *Baylisascaris transfuga* in moose calves. Branch support indicated by parsimony bootstrap above and Bayesian posterior probability below. Fig 3A. Parsimony analysis of the 12S rDNA sequences showing strict consensus of 2 equally parsimonious trees. Fig 3B. Parsimony analysis showing strict consensus of the *cox2* sequences which yielded four equally parsimonious trees (CI 0.86). Fig 3C. Parsimony analysis of the 28S rDNA sequences yielded one most parsimonious tree of length (CI 0.97). Fig. 3D. Parsimony analysis of the ITS rDNA sequences yielded one most parsimonious tree of length (CI 0.97).

*Phylogenetic analyses*: Parsimony analysis of the 12S rDNA sequences yielded two equally parsimonious trees of length 139 (CI 0.87). The strict consensus of these trees was identical in topology to the Bayesian consensus tree (Fig. 3A). The sequence of the larval *Baylisascaris* specimen from the moose was strongly supported (>99% bootstrap parsimony and Bayesian posterior probability, BPP) as part of an unresolved trichotomy including *B. transfuga*.

Parsimony analysis of the *cox2* sequences yielded four equally parsimonious trees of length 126 (CI 0.86). The strict consensus of these trees was less resolved that the Bayesian consensus tree (Fig. 3B), specifically the parsimony consensus tree was a polytomy for the clade consisting of *B. procyonis* and *B. columnaris*. The sequence from the larval *Baylisascaris* specimen received 100% support (bootstrap parsimony and BPP) as belonging to the clade containing *B. transfuga* and was weakly supported as sister to *B. transfuga* from a Canadian Grizzly bear.

Parsimony analysis of the 28S rDNA sequences yielded one most parsimonious tree of length 108 (CI 0.97). The topology of this tree was identical to the Bayesian consensus tree (Fig. 3C). The sequence from the larval *Baylisascaris* specimen received 100% support (bootstrap parsimony and BPP) as part of an unresolved trichotomy representing *B. transfuga*.

Parsimony analysis of the ITS rDNA sequences yielded one most parsimonious tree of length 314 (CI 0.97). The topology of this tree was identical to the Bayesian consensus tree (Fig. 3D). The sequence from the larval *Baylisascaris* specimen received 100% support (bootstrap parsimony and BPP) as part of an unresolved trichotomy representing *B. transfuga*.

**Fig. 4:**
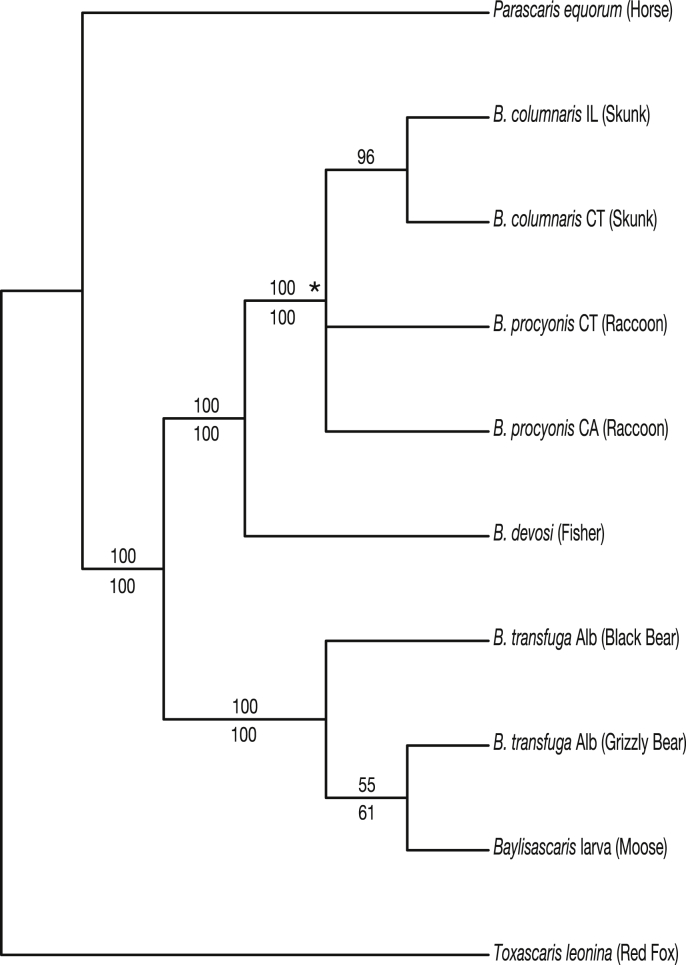
Parsimony analysis of the combined nuclear and mitochondrial genes yielded four equally parsimonious trees (CI 0.93). Strict consensus supported monophyly of *B. transfuga* and identity of the L3 recovered from moose.

Parsimony analysis of the combined dataset yielded four equally parsimonious trees of length 690 (CI 0.93). The strict consensus of these trees was less resolved than the Bayesian consensus tree (Fig. 4), specifically the parsimony consensus tree was a polytomy for the clade consisting of *B. procyonis* and *B. columnaris*. The sequence from the larval *Baylisascaris* specimen received 100% support (bootstrap parsimony and BPP) as part of the clade containing *B. transfuga* and was weakly supported as sister to *B. transfuga* from a Canadian grizzly bear.

*Distance comparisons*: Uncorrected pairwise percent sequence divergence for 12S rDNA and *cox2* showed no difference between the *Baylisacaris* larva from the moose and *B. transfuga* from a grizzly bear (Table 4). For these two genes, the next lowest pairwise divergence values for the larval specimen was to *B. transfuga* from a black bear. Uncorrected pairwise percent sequence divergence for 28S rDNA and ITS rDNA showed no differences between the *Baylisacaris* larva from the moose and *B. transfuga* from both grizzly and black bears (Table 5).

**Table 4:** Pairwise percent uncorrected sequence divergence (*p*-distance x 100) for the mitochondrial 12S region (listed first), followed by the *cox2* gene. Abbreviations indicate the sequence source: *Parascaris equorum* (Pa equ), *Baylisascaris columnaris* Illinois (B col IL), *B. procyonis* Connecticut (B pro CT), *B. procyonis* California (B pro CA), *B. columnaris* Connecticut (B col CT), *B. devosi* (B dev), *B. transfuga* Alberta black bear (B tra BB), *Toxascaris leonina* (T leo), *B. transfuga* Alberta grizzly bear (B tra GB), *Baylisascaris* larva from moose (B lar).

	P equ	B col IL	B pro CT	B pro CA	B col CT	B dev	B tra BB	T leo	B tra GB	B lar
P equ	–									
B col IL	15.3/9.3	–								
B pro CT	15.4/9.3	0.20/0.34	–							
B pro CA	15.3/9.1	0/0.52	0.20/0.52	–						
B col CT	15.1/9.6	0.20/0.34	0.4/0.70	0.20/0.52	–					
B dev	14.9/9.1	2.4/3.1	2.6/3.1	2.4/3.3	2.6/3.4	–				
B tra BB	15.0/9.1	8.2/7.6	8.4/7.6	8.2/7.7	8.2/7.9	8.0/8.8	–			
T leo	13.3/9.5	10.0/7.6	10.2/7.2	10.0/7.6	9.8/7.7	9.4/7.7	12.1/10.0	–		
B tra GB	14.8/8.9	8.0/7.6	8.2/7.6	8.0/7.7	8.0/7.9	7.8/8.8	0.20/0.34	11.9/10.0	–	
B lar	14.8/8.9	8.0/7.6	8.2/7.6	8.0/7.7	8.0/7.9	7.8/8.8	0.20/0.34	11.9/10.0	0/0	–

**Table 5:** Pairwise percent uncorrected sequence divergence (*p*-distance x 100) for the 28S region (listed first), followed by the ITS genes (ITS-1/5.8S/ITS-2). Abbreviations indicate the sequence source: *Parascaris equorum* (Pa equ), *Baylisascaris columnaris* Illinois (B col IL), *B. procyonis* Connecticut (B pro CT), *B. procyonis* California (B pro CA), *B. columnaris* Connecticut (B col CT), *B. devosi* (B dev), *B. transfuga* Alberta black bear (B tra BB), *Toxascaris leonina* (T leo), *B. transfuga* Alberta grizzly bear (B tra GB), *Baylisascaris* larva from moose (B lar).

	P equ	B col IL	B pro CT	B pro CA	B col CT	B dev	B tra BB	T leo	B tra GB	B lar
P equ	–									
B col IL	7.1/16.6	–								
B pro CT	7.1/16.6	0.09/0.24	–							
B pro CA	7.1/16.6	0.09/0.36	0/0.35	–						
B col CT	7.2/17.0	0.19/0.84	0.09/0.83	0.09/0.95	–					
B dev	7.0/16.0	0.66/2.6	0.56/2.6	0.56/2.7	0.66/3.2	–				
B tra BB	6.6/14.5	1.6/5.7	1.6/5.7	1.6/5.8	1.7/6.3	1.5/5.3	–			
T leo	6.9/23.6	3.7/22.2	3.6/22.1	3.6/22.1	3.7/22.6	3.5/21.6	3.7/20.9	–		
B tra GB	6.6/14.5	1.6/5.7	1.6/5.7	1.6/5.8	1.7/6.3	1.5/5.3	0/0	3.7/20.9	–	
B lar	6.6/14.5	1.6/5.7	1.6/5.7	1.6/5.8	1.7/6.3	1.5/5.3	0/0	3.7/20.9	0/0	–

## 4 Discussion

Correct or complete identification is important in revealing the potential routes of exposure and transmission along with the array of definitive and intermediate/paratenic hosts that may be involved in circulation for species of *Baylisascaris* at different geographic localities (e.g., Sapp et al., 2017). Risk for zoonotic infection would also be reflected based on the geographic distribution and transmission patterns for different species of *Baylisascaris*. Most prior reports and discussion of larval migrans (ocular, neural, visceral) attributable to species of *Baylisascaris* have suggested presumptive diagnoses of *B. procyonis*. Such diagnoses have often been presented in the absence of definitive evidence and absence of archived vouchers, even in regions where multiple species may be known to occur (e.g., reviewed in Kazacos, 2001; Gavin et al., 2005; Kazacos, 2016). Presumptive identification was most often linked to putative exposure and epidemiological observations consistent with the local occurrence of raccoons or less often skunks in the case of *B. columnaris* (Kazacos, 2001). This also parallels reports in Europe in regions where *B. procyonis* has been inadvertently introduced and established with the translocation of raccoons (e.g., Okulewicz and Buńkowska, 2009; Beltran-Beck et al., 2012).

### 4.1 Establishing identity

In the current Alaskan cases, the possibility of *B. procyonis* infection was eliminated based on the contemporary and historical distribution of raccoons as the primary definitive hosts for this ascaridoid (see Gehrt, 2003). The northern limit of natural range for raccoons in western North America extends to south coastal British Columbia (about 51° N) in the west and into southeast Alberta in the east (about 54° N) (e.g., MacDonald and Cook, 2009). The northern limits for distribution in western Canada have been expanding since the 1970's, but the core range remains over 1000 km to the south of localities in southcentral Alaska (e.g., Gehrt, 2003); a pertinent extralimital record is from Wood Buffalo National Park in northeastern Alberta (Soper, 1942). Although raccoons were released for possible introduction in areas around SE Alaska (Haida Gwaii and Alexander Archipelago) and adjacent to Kodiak Island at various times since the 1930's, none were translocated to central Alaska. Further, species of skunks, similar to raccoons, are not present in Alaska, and the range for striped skunk, *Mephitis mephitis* (Schreber), extends into southeastern Yukon and northern British Columbia, only approaching within 500 km. This geographic distribution effectively establishes the current absence of the largely host specific *B. columnaris* at higher boreal and subarctic latitudes (Rosatte and Larivière, 2003). Badgers as hosts of *B. melis*, are also well outside of the geographic range. Ursids as the hosts for *B. transfuga*, and mustelids as the hosts for *B. devosi* would appear to represent the sole endemic assemblages for circulation of these species of ascaridoids that are historically recognized and expected to have broad distributions in Alaska.

### 4.2 Morphological diagnostics

Based on the diameter of L3 in histological sections (maximum near 85 μm, Fig. 1), it was evident that larvae in moose differed substantially in diameter (width) at the midbody from those identified as *B. columnaris*, *B. devosi* or *B. procyonis* in prior studies (range about 45–65; or 55–70 μm in fully developed L3) (Sprent, 1953, 1955; Kazacos, 1986, 1997; Bowman, 1987); ranges for the diameter of L3 *B. transfuga* have apparently not been published. Despite these apparent differences, definitive diagnosis based solely on dimensions or structural attributes of the L3 among species of *Baylisascaris* remains elusive (e.g., Sapp et al., 2017). Through a rough process of elimination, however, L3 in moose may be attributable to *B. transfuga*. Further the greater diameter at the midbody, as demonstrated in our observations, clearly differentiates the L3 in moose from those of *Toxascaris leonina* (von Linstow, 1902) and *Toxocara cani*s (Werner, 1782) (e.g., Bowman, 1987), common ascaridoids of canid hosts in Alaska which may also be involved in cases of visceral larva migrans (Sprent, 1955; Jenkins et al., 2013; Rausch and Fay, 2011).

### 4.3 Molecular identification

Diagnosis of somatic and neurological infection by third-stage larvae among species of *Baylisascaris* continues to be challenging (Gavin et al., 2005; Bauer, 2013; Franssen et al., 2013; Kazacos, 2016; Sapp et al., 2017). The possibility of definitive molecular diagnosis for L3 in cases of larval migrans has not been universally available for species of *Baylisascaris* and serological tests have been non-specific (e.g., Boyce et al., 1988; Testini et al., 2011). Consequently, addressing or identifying the potential risk to public health posed by those species of *Baylisascaris* in circulation in North America (and globally) remains problematic (Franssen et al., 2013). Authoritative molecular identification and phylogenetic diagnosis including broad comparisons among congeners clearly established *B. transfuga* as the cause of neural infections in the sibling moose calves (Fig. 3, Fig. 4). There are apparently no prior records in ungulates for this species of *Baylisascaris*. Further explorations are necessary to refine the understanding of host and geographic range for *B. transfuga*. Broadening evidence indicates that the single nominal species as currently recognized encompasses a complex of distinct nematode taxa in North American bears (Sapp et al., 2017; Camp et al., submitted).

### 4.4 Neonatal or transuterine infections among ungulates

There has been limited evidence for neonatal infection linked to transuterine migration in potential ungulate paratenic hosts for species of *Baylisascaris* (Kazacos, 2016). The single prior report of natural neonatal infection in a lamb (*Ovis aries* L.) from Idaho lacked definitive species-level identification (Anderson, 1999).

Our current description and report of L3 *B. transfuga* clearly demonstrates a pattern of transuterine/*in utero* migration in the 1–3 day old moose siblings. Migration time to the brain is within 3–4 days post infection for *B. procyonis*, *B. columnaris* and *B. devosi*; 6–14 days to somatic sites for *B. transfuga.* A caveat in interpreting these observations is apparent as these time frames were based on experimental infection in mice and may not be representative (Sprent, 1952, 1953). Clinical onset of disease should mirror migration time to a considerable extent. The latter can vary substantially, however, with respect to host and parasite species along with patterns of exposure and dosage of infection (e.g., Sheppard and Kazacos, 1997; Sato et al., 2004; Sapp et al., 2016). Weaning time in moose is several months post parturition, although exploratory foraging may ensue within weeks of birth. These circumstances unequivocally indicate that infections were established in these neonates prior to birth. Vertical transmission would represent mobilization of larvae previously acquired and sequestered in the cow prior to or during pregnancy.

Among other ascaridoids, trans-placental migration was initially indicated for *Toxocara canis*, although this involved neonatal infection of canid definitive hosts (Sprent, 1954; Anderson, 2000). Further at the time of these initial studies during the 1950's it was known that L3 of ascaridoid species could be sequestered in somatic and other tissues as demonstrated for *B. devosi*, *B. columnaris*, *B. transfuga*, *Toxocara canis* and *Toxascaris leonina*. Neonatal infection in rodent and other mammalian paratenic hosts has also been documented experimentally for species of *Toxocara* Stiles, 1905 (e.g., Schnieder et al., 2011; Strube et al., 2013).

As established in experimental life cycle studies, one or two vertebrate paratenic (or intermediate?) hosts are required for completion of transmission among species of *Baylisascaris* in mammals (Sprent, 1952, 1953; Tiner, 1953; Anderson, 2000; Kazacos, 2001, 2016; Sapp et al., 2017). For *B. columnaris, B. procyonis* and *B. devosi*, migration in paratenic hosts (experimental mice) is rapid leading to widespread dissemination in the systemic circulation including sites in the somatic tissues and central nervous system within 3–4 days post infection (Sprent, 1952, 1953; Tiner, 1953). Maximum length and diameter of L3 are attained between 14 days and several months post infection and viable infective larvae may persist for extended periods of several months. Among these, *B. procyonis* and *B. columnaris* are more commonly associated with sites in the central nervous system of rodent paratenic hosts (Sprent, 1952, 1953; Tiner, 1953; Kazacos, 2001).

In infections of *B. transfuga*, similar migration rates were observed in experimental infections in rodent hosts, leading to permanent somatic infection by encysted L3 beginning near 8 days post infection (Sprent, 1952). Although viable larvae are encapsulated in various tissues, apparently this most often is associated with the wall of the intestine (Sprent, 1952, 1953). During extensive tissue migration, larvae of *B. transfuga* in experimental infections involving oral exposure had not been demonstrated uniformly in the central nervous system (Sprent, 1953, 1955; Anderson, 2000; Sato et al., 2004); L3 also may be involved in ocular infections (Papini et al., 1996). The time frames established for migration and development of L3 among species of *Baylisascaris* and the age of these sibling moose (at 1–3 days) is clearly consistent with a pattern of transuterine infection from the cow prior to parturition (the cow was not necropsied following death).

This is the first instance of definitive species-level identification of neonatal infection attributed to *B. transfuga*, or any species of *Baylisascaris,* in a naturally infected putative ungulate paratenic host (e.g., Kazacos, 2001, 2016). In the current cases verminous encephalitis attributed to *B. transfuga*, contributed directly to the debilitated condition of these sibling moose and in the absence of euthanasia, these calves are unlikely to have survived in the wild. Anderson (1999) reported a fatal neurological infection attributed to a species of *Baylisascaris* in a 2–3 day old domestic lamb from Idaho, however, the causative agent could not be determined. Possible involvement of *B. procyonis*, *B. melis* or *B. columinaris* was suggested, but *B. transfuga* was not included in this discussion. *Baylisascaris* L3 are known from a considerable array of mammalian hosts either from natural infections or experimentally (e.g., Kazacos, 2001, 2016), however, infections in ungulates are virtually unknown. Demonstrating that infections are determined by opportunity, exposure and biological capacity of parasites Sato et al. (2005) reported multiple cases of fatal neurological disease among Japanese macaques (*Macaca fuscata* Blythe) under open zoo-park conditions. The primate colony was maintained in proximity to 11 North American black bears known to be infected with *B. transfuga.* Raccoons also adjacent to the colony were not known to be infected with *B. procyonis.* Species-level identification was not possible, although the nematodes were attributed tentatively to *B. transfuga*.

### 4.5 Broader implications for Baylisascaris

Assuming the cryptic nature of infections, the significance of this pattern of larval development in the life cycle of *B. transfuga* and potential transmission among moose (and other artiodactyls) and bears would remain to be established. The complete life cycle for *B. transfuga* in free-ranging wild hosts has not been elucidated and could involve direct transmission through ingestion of larvated eggs, or dissemination through infected mammalian paratenic hosts (Bauer, 2013; Sapp et al., 2017). The latter pathway would be consistent with life history patterns currently known among congeners (e.g., Sprent, 1954, 1968; Anderson, 2000). Among black bears the diet is dominated by vegetation and vegetable resources, and among these omnivores, consumption of animals usually involves carrion or is opportunistic (Pelton, 2003). In contrast, brown bears are considerably more carnivorous, and predation on ungulates may be seasonally important especially for populations in the continental interior of North America (Schwartz et al., 2003). Such was reflected in observations (EPH) of a large brown bear stalking and hunting moose calves along the Igloo Creek Canyon in Denali Park, Alaska in June 2016. Rodents, especially the prolific and abundant Arctic ground squirrels [*Urocitellus parryii* (Richardson)] are also important sources of food at high latitudes for brown bears.

Life history and involvement of paratenic hosts across a broader assemblage of mammals, from rodents to ungulates, in the transmission of *B. transfuga* remains undefined (e.g., Tiner, 1953; Sprent, 1954; Testini et al., 2011; Bauer, 2013; Sapp et al., 2017). Further, the nature of abortion or neonatal mortality in moose, involving larval *B. transfuga*, also remains to be revealed and although logistically challenging to demonstrate, should be considered in diagnosis. Migration by developing larvae and L3 late in gestation may result in infections of the central nervous system as demonstrated in our study. In contrast, migration early in gestation could be a contributing factor in spontaneous abortion in some cases, although, again, evidence is lacking. Infections of neonatal ungulates, however, could be a factor in completion of transmission to carnivore definitive hosts, in this case either brown bear or black bear, which are common and widespread in Alaska and south into the temperate zone of North America.

Discovery of fatal neurological infections by *B. transfuga* in naturally infected mammalian hosts serves to demonstrate the potential for zoonotic infection, as widely established for *B. procyonis* in other regions and where raccoon definitive hosts are abundant (Kazacos, 1997, 2001; 2016; Papini et al., 1996; Bauer, 2013; Testini et al., 2011; Sapp et al., 2017). More importantly in zones of sympatry for multi-species assemblages of *Baylisascaris* across the Holarctic region presumptive identification of *B. procyonis* in cases of neurological larval migrans must be considered with caution. Where possible presumptive identification of *B. procyonis* or other species of *Baylisascaris* should be confirmed and accompanied by molecular sequence comparisons established in a phylogenetic context (e.g., Camp et al., submitted).

Northward expansion, associated with habitat and environmental change linked to climate warming could lead to changing distribution for raccoons and skunks and therefore, as a consequence, increasingly broader distributions for *B. procyonis* and *B. columnaris* (e.g., Hoberg et al., 2008; Hoberg, 2010; Jenkins et al., 2013). Patterns of geographic colonization by these assemblages would also be predicted to directly influence helminth diversity. Potential lag times for invasion by parasites including *B. procyonis* may coincide with shifting abundance of definitive hosts on the periphery of expanding geographic ranges (Hoberg and McGee, 1982; Hoberg et al., 2017). The expectation or anticipation of range shifts (either increasing or decreasing) for potential zoonotic pathogens and an array of hosts establishes a priority for definitive documentation of diversity and species. Baselines for identification and biodiversity can lead to surveillance and targeted monitoring to explore and anticipate the outcomes of environmental perturbation over time (Brooks et al., 2014).
